# Genetic contributions to the expression of acquired causes of cardiac hypertrophy in non-ischemic sudden cardiac death victims

**DOI:** 10.1038/s41598-021-90693-7

**Published:** 2021-05-27

**Authors:** Lauri Holmström, Katri Pylkäs, Anna Tervasmäki, Juha Vähätalo, Katja Porvari, Lasse Pakanen, Kari S. Kaikkonen, Juha S. Perkiömäki, Antti M. Kiviniemi, Risto Kerkelä, Olavi Ukkola, Robert J. Myerburg, Heikki V. Huikuri, Juhani Junttila

**Affiliations:** 1 Research Unit of Internal Medicine, Medical Research Center Oulu, University of Oulu and Oulu University Hospital, PO Box 5000, 90014 Oulu, Finland; 2 Laboratory of Cancer Genetics and Tumor Biology, Cancer and Translational Medicine Research Unit and Biocenter Oulu, University of Oulu, Oulu, Finland; 3 Department of Forensic Medicine, Research Unit of Internal Medicine, Medical Research Center Oulu, University of Oulu, Oulu, Finland; 4 Forensic Medicine Unit, National Institute for Health and Welfare (THL), Oulu, Finland; 5 Research Unit of Biomedicine, University of Oulu, Oulu, Finland; 6 Division of Cardiology, University of Miami Miller School of Medicine, Miami, FL USA

**Keywords:** Cardiovascular genetics, Cardiac hypertrophy

## Abstract

The contribution of genetic variants to non-ischemic sudden cardiac death (SCD) due to acquired myocardial diseases is unclear. We studied whether SCD victims with hypertension/obesity related hypertrophic myocardial disease harbor potentially disease associated gene variants. The Fingesture study has collected data from 5869 autopsy-verified SCD victims in Northern Finland. Among SCD victims, 740 (13%) had hypertension and/or obesity as the most likely explanation for myocardial disease with hypertrophy and fibrosis. We performed next generation sequencing using a panel of 174 cardiac genes for 151 such victims with the best quality of DNA. We used 48 patients with hypertension and hypertrophic heart as controls. Likely pathogenic variants were identified in 15 SCD victims (10%) and variants of uncertain significance (VUS) were observed in additional 43 SCD victims (28%). In controls, likely pathogenic variants were present in two subjects (4%; p = 0.21) and VUSs in 12 subjects (25%; p = 0.64). Among SCD victims, presence of potentially disease-related variants was associated with lower mean BMI and heart weight. Potentially disease related gene variants are common in non-ischemic SCD but further studies are required to determine specific contribution of rare genetic variants to the extent of acquired myocardial diseases leading to SCD.

## Introduction

Left ventricular hypertrophy (LVH) is a major risk factor for morbidity and mortality in Western societies, in association with both coronary artery disease (CAD) and non-ischemic heart diseases^1–3^. In association with the growing epidemic of obesity in the Western world, LVH is becoming increasingly recognized as a consequence of obesity, and may also be associated with myocardial fibrosis. Obesity-related LVH is presumed to be caused by an interaction between increased cardiac workload due to the excess body weight and comorbid hypertension^4^. Pathologic hypertrophy has a strong association with the incidence of sudden cardiac death (SCD), and the majority of ischemic and non-ischemic SCD victims express hypertrophy with different patterns of fibrosis at autopsy^2, 3, 5^.

Among young SCD victims, hypertrophic cardiomyopathy (HCM) arrhythmogenic cardiomyopathy (ACM), primary myocardial fibrosis (PMF) and dilated cardiomyopathies (DCM) are common findings at autopsy, but hypertension and obesity related myocardial diseases are more common in subjects over 40 years of age^3, 6^. Considering the wide spectrum of phenotypic expression of inherited diseases, it is reasonable to speculate on the generalization that common acquired causes for cardiac hypertrophy may interact with genetic variants, leading to disease progression and ultimately to a risk for life-threatening arrhythmias.

In this study, our goal was to determine whether non-ischemic myocardial diseases with LVH/fibrosis at autopsy after SCD and hypertension and/or obesity as the apparent triggers for LVH, may associate with genetic variants in arrhythmia candidate genes.

## Methods

### The Fingesture study

**Figure 1:**
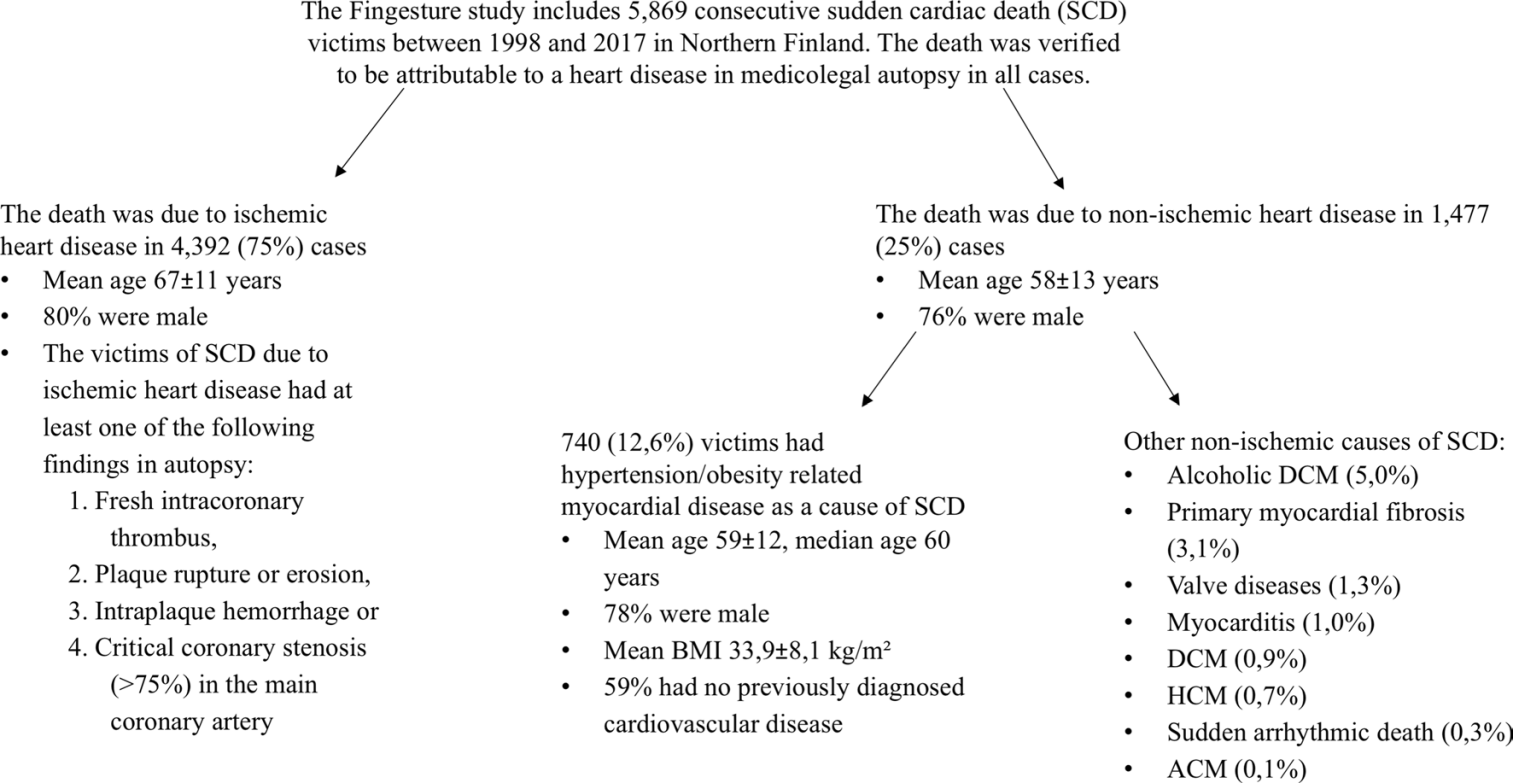
Description of autopsy findings in the Fingesture study. Continuous data is presented as mean ± standard deviation. *ACM* arrhythmogenic cardiomyopathy, *BMI* Body Mass Index, *DCM* dilated cardiomyopathy, *HCM* hypertrophic cardiomyopathy, *SCD* Sudden cardiac death.

The study population is derived from the Fingesture study, which has gathered autopsy and clinical record data from 5869 consecutive SCD victims from the Oulu University Hospital District, a defined geographical area in northern Finland since 1998. The detailed study protocol has been previously published^3^. Briefly, a medicolegal autopsy was performed on all SCD victims in the National Institute for Health and Welfare, Oulu, Finland and at the Department of Forensic Medicine, University of Oulu, Oulu, Finland, by experienced forensic pathologists, each performing over 100 autopsies/year, using contemporary guidelines for diagnosing the cause of death. Finnish law requires medicolegal autopsy to be performed if the death is not due to a previously identified disease, a victim who was not treated by physician during his/her last illness, or if the death was otherwise unexpected. Therefore, Finland has the highest autopsy rates following SCD in Western societies^7^ and the Fingesture study includes a majority of victims of unexpected SCD (estimated to be 60% of all sudden deaths)^8^ in the defined area. Meticulous cardiac investigations were performed on all victims, including macroscopic dissection and investigation of myocardium, coronary arteries and valves, and histological samples taken from 3 to 5 sections of the heart. All causes of SCD in the Fingesture study during 1998–2017 are provided in Fig. 1.

### SCD victims in the present study

We selected 151 non-ischemic SCD victims who were found to have non-specific LVH and fibrosis at autopsy without diagnostic criteria for HCM, such as myocyte disarray or asymmetric septal hypertrophy. The inclusion criteria included cardiac hypertrophy at autopsy determined by a heart weight greater than the predicted value based on body surface area. In addition to cardiac hypertrophy, a clinical history of hypertension and/or autopsy findings related to hypertension (e.g. sclerotic renal arterioles) were characteristic of subjects with a hypertensive etiology. Obesity-induced myocardial disease included obesity with a BMI > 30, with excessive epicardial fat with or without LV dilatation^3^. Study subjects had no clinical history of CAD and coronary arteries had no stenosis > 50% or active plaques at autopsy. Patients with developmental or acquired aortic stenosis were not included.

DNA samples of the SCD victims were isolated from formalin fixed and paraffin embedded myocardial tissue samples taken at autopsy. We carried out genetic studies in the 151 of 740 individuals (20%) with SCD who met the criteria for acquired LVH due to hypertension or obesity, and whose DNA passed the quality control for further analysis.

### Control subjects

The control study population is from the OPERA (Oulu Project Elucidating Risk of Atherosclerosis) project, which is an epidemiological, population-based study designed to address the risk factors and disease endpoints of atherosclerotic cardiovascular disease. The study population has been previously described in detail^9^. The study population at baseline consisted of a hypertensive cohort (261 men and 258 women) and a control cohort (259 men and 267 women) from the city of Oulu, Finland. Subjects were 40–59 years old at the time of recruitment. Subjects were recruited during December 1990 to March 1993, and after 20 years, the subjects were called for a follow up visit. Echocardiographic measurements were performed in both baseline and after 20 years of follow-up. Forty-four hypertensive subjects (8%) had severely abnormal left ventricle mass index (LVMI; ≥ 122 g/m^2^ for women and ≥ 149 g/m^2^ for men) at both baseline and follow-up measurements. An additional 76 hypertensive subjects had abnormal LVMI in baseline, but not at follow-up measurement. For DNA analysis we chose subjects with hypertensive hypertrophic heart disease at follow-up without SCD. At first we chose 44 hypertensive subjects with severely abnormal LVMI in both measurements, and subsequently 4 subjects with severely abnormal LVMI at baseline but not at follow-up to fulfill required 48 samples in the TruSight Cardio gene panel kit. DNA of the study subjects was extracted from white blood cells taken during follow-up visit.

### DNA sequencing

DNA sequencing methods have been described earlier^10^. The TruSight Cardio gene panel kit, composed of 174 genes with associations with inherited cardiac conditions most affected by a genetic predisposition (http://support.illumina.com/downloads/trusight-cardio-product-files.html), was used for library preparation (Illumina, San Diego, CA; Table 1). Samples were bead purified with Agencourt AMPure XP beads (Beckman Coulter Life Sciences, Indianapolis, IN). The quality of the samples selected for next generation sequencing (NGS) was confirmed with quantitative polymerase chain reaction–based formalin fixed paraffin-embedded quality control kit (Illumina), and the samples passing quality control based upon a quantitative polymerase chain reaction ΔCq value ≤ 2.3, were selected for gene panel sequencing with NextSeq550 platform (Illumina). Within the BaseSpace Genomics computing environment (Illumina), BWA Enrichment (BWA Genome Aligner Software and the GATK Variant Caller) was used for sequence alignment and variant calling; VariantStudio for annotation, filtering, and classification of the variants; and Integrative Genomics Viewer for data visualization to exclude falsely annotated variants and sequencing artifacts. All variants classified as likely pathogenic and with read depth < 50 were confirmed by Sanger Sequencing (ABI3130xl, Applied Biosystems, Foster City, CA).

**Table 1:** Cardiac structure- and function-related genes sequenced in the panel classified by disease associations.

HCM related	*ACTC1, ****ACTN2****, ****ANKRD1****, CALR3, ****CAV3****, ****CSRP3****, JPH2, MYBPC3, ****MYH6****, ****MYH7****, MYL2, MYL3, ****MYLK2****, MYO6, MYOZ2, MYPN, ****NEXN****, PDLIM3, PLN, PRKAG2, ****TCAP****, TNNC1, TNNI3, TNNT2, TPM1, TRIM63, ****VCL***
ACM related	***DES****, ****DSC2****, ****DSG2****, ****DSP****, JUP, ****LMNA****, ****PKP2****, PLN, ****RYR2****, SCN5A, TGFB3, TMEM43*
DCM related	***ABCC9****, ****ACTN2****, ACTC1, ****ANKRD1****, BAG3, CRYAB, ****CSRP3****, ****DES****, DMD, ****DSG2****, EYA4, GATAD1, ****LAMA4****, ****LDB3, LMNA****, MYBPC3, ****MYH6****, ****MYH7****, MYPN, ****NEXN****, PLN, ****RBM20****, SCN5A, ****SGCD****, TAZ, ****TCAP****, TMPO, TNNC1, TNNI3, TNNT2, TPM1, ****TTN****, ****VCL****, ZBTB17*
LVNC related	***DTNA****, ****LDB3****, ****LMNA****, MIB1, MYBPC3, ****MYH7****, PRDM16, TAZ, TNNT2, TPM1*
Metabolic disorders and syndromes with cardiac diseases and congenital heart defects	*ALMS1, BRAF, CBL, COX15, CRELD1, DNAJC19, DOLK, FXN, GAA, GLA, HFE, HRAS, JAG1, KRAS, LAMP2, MAP2K1, MAP2K2, NKX2-5, NODAL, NOTCH1, NRAS, PTPN11, RAF1, SCO2, SDHA, SHOC2, SMAD4, SOS1, TBX3, TBX20, TBX5, TTR, ZIC3*
Arrhythmic disorders	*AKAP9, ANK2, CACNA1C, CACNA2D1, CACNB2, CALM1, ****CASQ2****, ****CAV3****, DPP6, GJA5, GPD1L, HCN4, KCNA5, KCND3, KCNE1, KCNE2, KCNE3, KCNH2, KCNJ2, KCNJ5, KCNJ8, KCNQ1, NPPA, RANGRF, ****RYR2****, SCN1B, SCN2B, SCN3B, SCN4B, SCN5A, SNTA1, TRDN, TRPM4*
Dyslipidemia	*ABCG5, ABCG8, APOA5, APOB, APOC2, APOE, CETP, GPIHBP1, LDLR, LDLRAP1, LMF1, LPL, PCSK9, SREBF2*
Aortopathies/EDS	*ACTA2, COL3A1, COL5A1, COL5A2, EFEMP2, ELN, FBN1, FBN2, MYH11, MYLK, SLC2A10, SMAD3, TGFB2, TGFB3, TGFBR1, TGFBR2*
Muscular dystrophies/myopathies	*ACTA1, BAG3, EMD, FHL1, FKRP, FKTN, LAMA2, RYR1, SEPN1, SGCB, ****SGCD****, SGCG, SLC25A4, TMEM43*
Other	*APOA4, CBS, CREB3L3, ****CTF1****, FHL2, GCKR, HADHA, HSPB8, ILK, KLF10, LTBP2, MURC, PRKAR1A, SALL4, TXNRD2, ZHX3*

Genes in bold had potentially disease related variants in sudden cardiac death victims.

*ACM* arrhythmogenic cardiomyopathy, *DCM* dilated cardiomyopathy, *EDS* Ehlers-Danlos syndrome, *HCM* hypertrophic cardiomyopathy, *LVNC* left ventricular non-compaction cardiomyopathy.

### Variant analysis

The SCD victims were derived from the total of 151 qualifying cases and resulted in mean read depth of × 1078 per sample. On average, 99.4% of the captured region (0.572 Mb) was covered at least by 20 reads and 99.0% at least by 50 reads for the analyzed samples. In the control group, a mean read depth of × 1048 per sample was obtained, 99.6% and 99.4% of the captured region was covered at least by 20 and 50 reads, respectively. All variants with a potential effect on protein were selected for analysis and filtered further according to their prevalence in dbSNP or Exome Aggregation Consortium database by excluding variants with minor allele frequency (MAF) > 0.01 among Finnish subjects. Further assessments for pathogenicity were based on American College of Medical Genetics (ACMG) consensus guidelines^11^. Likely benign variants and rare missense variants in *TTN*-gene were excluded from the results. Variants that did not meet the criteria for likely benign, were further classified as either (1) Pathogenic, (2) Likely pathogenic or as (3) Variant of uncertain significance (VUS), based on ACMG-guidelines using previous literature, population frequency (gnomAD and The Sequencing Initiative Suomi (SISu) databases), in silico algorithms (SIFT, PolyPhen), ClinVar database and the Human Gene Mutation Database.

### Statistical methods

Continuous variables are expressed as mean ± SD. Two-sided t-test and χ^2^-test were used to compare continuous and categorical characteristics between groups of interest, respectively. If skewed distribution (|skewness| > 1, heart weight, BMI) was encountered in continuous variables, the variable was transformed into natural logarithm and distributions were thereafter verified as Gaussian. If a specific variant appeared in multiple study subjects, statistical significance, odds ratios (OR) and 95% confidence intervals (CI) were assessed using χ^2^ test with two-sided p value (Fisher's Exact Test). The Sequencing Initiative Suomi (SISu) database was used as a control group, including data on genetic variants from 10,490 exome sequenced Finnish citizens (URL: http://sisuproject.fi). All analyses were performed with the Statistical Package for Social Studies version 21.0 (SPSS Inc, Chicago, IL). All p values are 2-sided and values < 0.05 were considered as significant.

The study complies with the Declaration of Helsinki, and the Ethics Committee of Northern Ostrobothnia Hospital District and the National Authority for Medicolegal Affairs (Valvira) approved the study. Consent from next of kin was waived by the Ethics Committee since according to the Finnish law, medicolegal autopsy does not require consent.

## Results

Mean age of SCD victims was 54 ± 10 years, 82% were male and 50% had prior cardiac disease diagnosis. The presumed etiological basis for LVH was hypertension in 78 of the SCD victims (52%), and obesity was considered the most likely cause for LVH in the remaining 73 subjects (48%). In total, potentially disease-related variants (likely pathogenic or VUS) were present in 57 (38%) of the 151 SCD subjects. All variants were heterozygous. Likely pathogenic variants were present in 15 (10%) subjects. Seventy-six non-synonymous variants with MAF < 0.01 in SCD victims were classified as likely benign. There was no difference in prevalence of associated genetic variants between hypertension-associated versus obesity-associated SCD victims (40% vs 36% respectively; p = 0.62). Most of the potentially disease related variants were in HCM, ACM and DCM-related genes. SCD victims with likely pathogenic or uncertain variants had lower mean BMI (30.3 ± 5.9 kg/m^2^ vs. 33.4 ± 7.2 kg/m^2^; p = 0.003) and heart weight (528 ± 104 g vs. 557 ± 97 g; p = 0.05) than those without variants, but there was no statistically significant difference in the prevalence of patchy/diffuse myocardial fibrosis. Characteristics of SCD victims are presented in Table 2.

**Table 2:** Characteristics of the study subjects classified by the presence of rare gene variants (likely pathogenic or variants of uncertain significance).

Characteristic	All subjects (n = 151)	Subjects with relevant variants (n = 57)	Subjects without relevant variants (n = 94)	P value
Age, mean ± SD (range), years	54 ± 10 (20–89)	55 ± 12 (20–89)	54 ± 9 (35–78)	0.38
Male gender, %	82.1	82.8	81.7	0.87
Prior cardiac disease diagnosis, %	49.7	47.3	51.1	0.65
Heart failure, %	8.3	9.3	7.8	0.76
Patchy/diffuse fibrosis at autopsy, %	88.1	91.4	86.0	0.32
Heart weight at autopsy, mean (SD), g	546 ± 100	528 ± 104	557 ± 97	0.05
BMI, mean (SD), kg/m^2^	32.2 ± 6.9	30.3 ± 5.9	33.4 ± 7.2	0.003

*BMI* body mass index, *SD* standard deviation.

### Likely pathogenic variants in SCD victims

One subject carried two likely pathogenic variants (p.Asp312Asn in *DES* and c.346-2A>G in *MYH6)* and the other 14 carried one each*.* Variants previously associated with inherited cardiomyopathies were identified, including variants associated with HCM patients (p.Gln1065His in *MYH6*^12^*,* p*.*Gln9Arg in *ACTN2*^13^, p.Met982Thr in *MYH7*^14^ and p.Arg153His in *TCAP*^15^), ACM patients (p.Ala372Pro in *PKP2*^16^ and p.Val392Ile in *DSG2*^17^), or DCM patients (p.Asp312Asn in *DES*^18^ and p.Arg66Gln in *ANKRD1*^19^). Two variants were also present in PMF subjects in our previous study (p.Met982Thr in *MYH7* and p.Ala372Pro in *PKP2*)^10^. Also, p.Thr78Met in conserved residue of *CAV3* is previously associated with functional channelopathy^20^. Two subjects carried the p.Thr78Met variant and another had a pre-mortem ECG with normal QT interval, normal QRS duration, and no Brugada pattern in right precordial leads. One subject carried p.Gln168His variant in *LMNA,* which has no population frequency in Finland, was predicted to be damaging by in silico algorithm and located close to previously established pathogenic *LMNA* variant p.Arg166Pro.

Three likely pathogenic variants were described in multiple SCD victims; p.Arg66Gln in *ANKRD1* (n = 3), p.Val392Ile in *DSG2* (2) and p.Thr78Met in *CAV3* (2). Among the carriers of these variants, the difference in prevalence of affected carriers, when compared to Finnish control population, was statistically significant for p.Arg66Gln (3/151 vs. 25/10,489; p = 0.007, OR = 8.5, 95% CI 2.5–28.4) and in p.Val392Ile (2/151 vs. 19/10,489; p = 0.04, OR = 7.4, 95% CI 1.7–32.0). Higher prevalence of *CAV3* variant among SCD victims was however, not statistically significant (2/151 vs. 42/8,540; p = 0.18, OR = 2.7, 95% CI 0.7–11.3). Characteristics of SCD victims with likely pathogenic variants are described in Table 3.

**Table 3:** Summary of likely pathogenic variants among sudden cardiac death victims with presumed acquired cardiac disease.

Mutated gene	Subject no	Decade of life, gender	Presumed etiology of LVH	Heart weight, g	Myocardial fibrosis at autopsy	Nucleotide change	Effect on protein	Predicted effect	NGS coverage	gnomAD > 10,000 Finnish controls MAF	SISu > 10,000 Finnish controls MAF	ACMG score^16^	ClinVar adjudication
*ABCC9*	1	60M	Hypertension	476	Moderate patchy	565C>T	Arg189Ter	Truncating	175	0.0011	0.0008	PVS1 + PM4 + PP3	N/A
*ACTN2*	2	50M	Hypertension	427	Scattered mild	26A>G	Gln9Arg	Missense	261	0.0002	0.0001	PS3 + PP2 + PP4 (DCM, HCM)	Conflicting
*ANKRD1*	3	50M	Hypertension	446	Scattered mild	197G>A	Arg66Gln	Missense	369	0.0015	0.0012	PS4 + PP3 + PP4 (DCM)	Conflicting
*ANKRD1*	4	70M	Obesity	440	Scattered mild	197G>A	Arg66Gln	Missense	353	0.0015	0.0012	PS4 + PP3 + PP4 (DCM)	Conflicting
*ANKRD1*	5	30M	Obesity	525	Moderate patchy	197G>A	Arg66Gln	Missense	160	0.0015	0.0012	PS4 + PP3 + PP4 (DCM)	Conflicting
*CAV3*	6	50M	Hypertension	463	Scattered mild	233C>T	Thr78Met	Missense	181	0.0032	0.0025	PS1 + PP3 + PP4 (LQTS)	Conflicting
*CAV3*	7	50M	Obesity	411	Scattered mild	233C>T	Thr78Met	Missense	46^a^	0.0032	0.0025	PS1 + PP3 + PP4 (LQTS)	Conflicting
*DES*	8	60M	Hypertension	476	Scattered mild	934G>A	Asp312Asn	Missense	248	Not detected	Not detected	PS3 + PM1 + PM2 + PP2 + PP3 + PP4 (DCM) + PP5	VUS
*DSG2*	9	80F	Hypertension	529	Moderate patchy	1174G>A	Val392Ile	Missense	58	0.0009	0.0009	PS4 + PP2 + PP3 + PP4 (ACM)	Conflicting
*DSG2*	10	70F	Hypertension	620	Some fibrosis	1174G>A	Val392Ile	Missense	119	0.0009	0.0009	PS4 + PP2 + PP3 + PP4 (ACM)	Conflicting
*LMNA*	11	50M	Hypertension	454	Moderate patchy	504G>C	Gln168His	Missense	77	Not detected	Not detected	PM1 + PM2 + PP2 + PP3	VUS
*MYH6*	8	60M	Hypertension	476	Scattered mild	346-2A>G		Affects canonical splicing	112	Not detected	Not detected	PVS1 + PM2	VUS
*MYH6*	12	50M	Obesity	673	Some fibrosis	3195G>C	Gln1065His	Missense	319	0.0017	0.0017	PS1 + PM1 + PP3 + PP4 (HCM)	Conflicting
*MYH7*	13	50M	Obesity	472	Moderate patchy	2945 T>C	Met982Thr	Missense	95	0.0005	0.0005	PS1 + PM1 + PP2 + PP3 + PP4 (HCM)	Benign
*PKP2*	14	50M	Obesity	651	Moderate patchy	1114G>C	Ala372Pro	Missense	23^a^	0.0023	0.0023	PM6 + PP2 + PP3 + PP4 (ACM)	Conflicting
*TCAP*	15	40M	Obesity	530	Some fibrosis	458G>A	Arg153His	Missense	131	0.0022	0.0019	PS1 + PP1 + PP2 + PP4 (HCM)	Conflicting

*ACMG* American college of molecular genetics, *ACM* arrhythmogenic cardiomyopathy, *DCM* dilated cardiomyopathy, *HCM* hypertrophic cardiomyopathy, *LQTS* long QT syndrome, *LVH* left ventricle hypertrophy, *MAF* minor allele frequency, *NGS* next generation sequencing, *VUS* variant of uncertain significance.

^a^Verified by Sanger sequencing.

### Variants of uncertain significance in SCD victims

Forty-nine (32%) SCD victims carried uncertain variants. These variants were considered as potentially disease-associated, rather than as likely benign due to previous reports in cardiomyopathy patients, location in a mutational hot spot, and/or prediction to be damaging by in silico algorithms. Uncertain variants were predominantly missense by type but one was predicted to have effect on canonical splicing (c.11254 + 2T>C in *TTN*). We classified this variant as VUS because loss of function variants in *TTN* gene are relatively common in general population (1–2%), and disease causing variants locate typically in the A-band or in highly expressed exon whereas our variant did not^21^. Seven subjects carried both likely pathogenic variants and VUSs, and 5 subjects carried multiple dissimilar uncertain variants. Eight VUSs were concordant in multiple SCD victims. The uncertain variants are presented in the Supplementary Table 1.

### Control subjects

**Figure 2:**
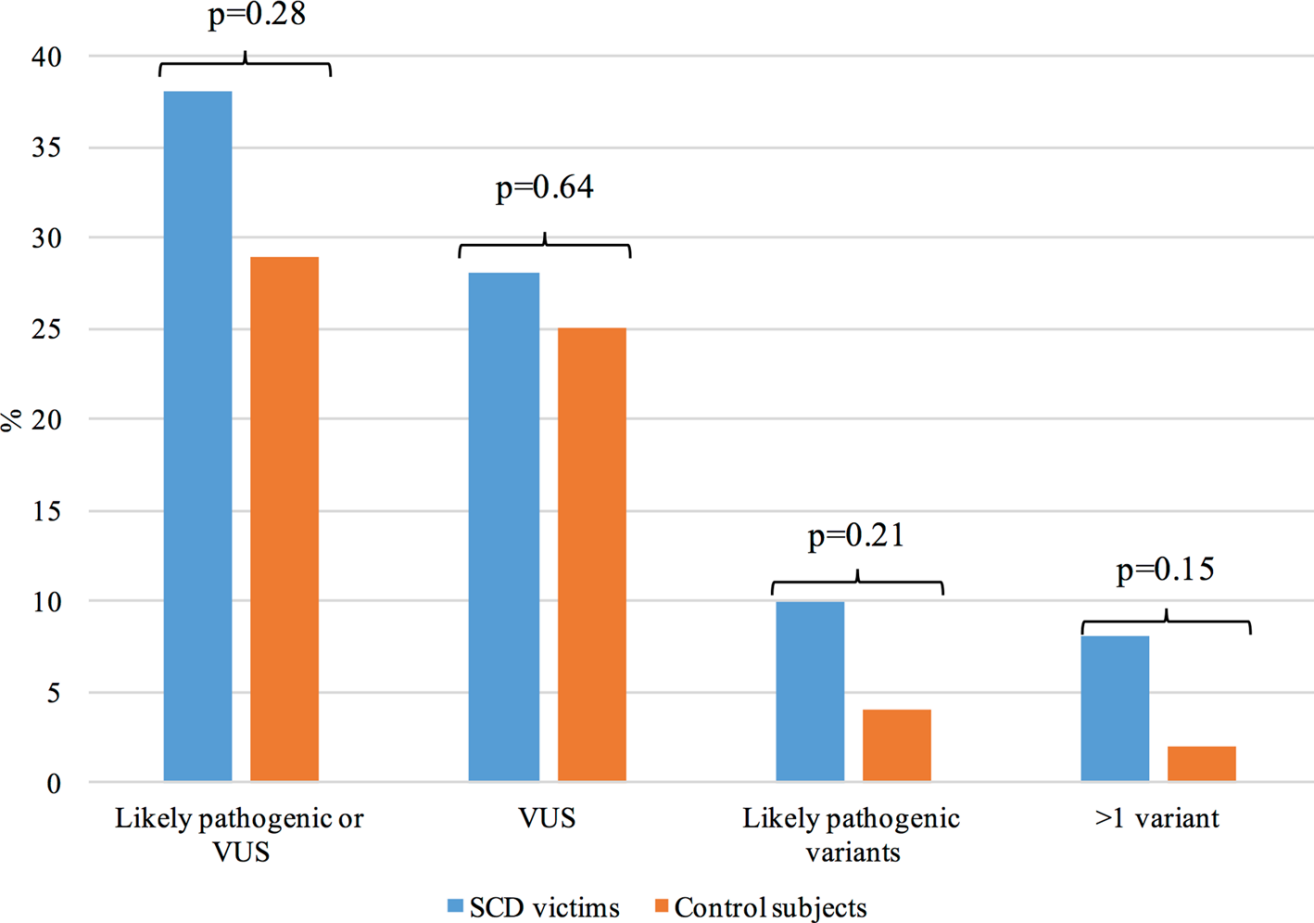
Prevalence of potentially disease related gene variants among SCD victims and control subjects.

Two control subjects with hypertension and hypertrophic heart (4%) carried likely pathogenic variants; Gly853Argfs (c.2555dupT) in *MYBPC3,* and Arg219Ter (c.566C>T) in *LDB3*. These variants were classified as likely pathogenic due to their absence from, or a very low frequency in, the general population and predicted loss of function effect. Additional 12 (25%) control subjects carried uncertain variants, which are presented in Supplementary Table 2. One subject carried two uncertain variants. The differences in the prevalence of likely pathogenic variants or VUSs were not statistically significant between SCD victims and control subjects (Fig. 2).

## Discussion

Our systematic collection of almost 6000 consecutive autopsied SCD victims have shown that non-ischemic causes of SCD are not limited to young victims and monogenic etiologies, but constitutes also notable burden of SCD cases in victims over 40 years with acquired etiologies. In this study, 10% of SCD victims with presumably acquired non-ischemic cardiac hypertrophy at autopsy harbored likely disease-related rare genetic variants, and additional 28% carried VUSs. Among 48 control subjects with hypertension-associated LVH over 20 years of follow-up, two carried likely pathogenic variant (4%), and additional 25% carried VUSs. Although the prevalence of likely disease-causing variants was less than one-half of that observed in the SCD victims, the difference was not statistically significant. Of interest, observed likely pathogenic variants in control subjects may also have an effect on the cardiac hypertrophy.

Previous studies investigating the genetic background of SCD due to non-specific structural cardiac diseases have reported conflicting results. Recent study from Lahrouchi et al. reported likely pathogenic variant in 1/29 SCD victim with autopsy findings of uncertain significance, whereas Hertz et al. reported rare variants with likely functional effects in 29% of SCD victims with non-diagnostic structural changes at autopsy^22, 23^. Variable results may be explainable by different MAF thresholds for variant analysis. In comparison to aforementioned studies, our study subjects were not restricted to young SCD victims and had hypertension/obesity related myocardial disease at autopsy. Although our study subjects were substantially younger than general SCD patients with CAD, they were on average older than those dying suddenly associate with a typical HCM phenotype^24^.

SCD victims with possibly disease related variants had lower mean BMI and heart weight in comparison to those without. Lower BMI is understandable by the view that high mismatch between acquired factors and the magnitude of cardiac disease may denote genetic predisposition in the disease development. Reasons for lower heart weight are not that obvious. Taken into notice that there was no notable difference in the prevalence of substantial myocardial fibrosis at autopsy, lower heart weight in subjects with relevant variants may represent higher fibrosis/hypertrophy-ratio. This may be in concordant with previous studies which have suggested that patients with genetic predisposition to LVH (HCM) have more myocardial fibrosis that those with hypertensive LVH^25, 26^.

Many potentially disease related variants in our study had conflicting interpretations of their pathogenicity in ClinVar. These variants have properties that support pathogenic nature (e.g. descriptions in disease patients, in silico predictions, conservative residues) and their conflicting interpretation is mostly due to relatively high MAF, which is inconsistent with inherited cardiomyopathies, and non-definitive causal association of suspected genes with HCM, DCM or ACM. However, our SCD victims did not present with these cardiomyopathies, but rather with acquired myocardial diseases that had common structural abnormalities. Inherited cardiomyopathies are common in young SCD victims, but the overall incidence of SCD in young people is low. As seen in the Fingesture study, inherited cardiomyopathies only account for a minority of SCD cases due to non-ischemic myocardial diseases in general population (Fig. 1). Moreover, the genetic underpinnings of non-ischemic SCDs are probably much more diverse than the very rare highly pathogenic variants that cause Mendelian diseases.

Altogether, we assume that the variants in our study may not be the main cause of myocardial diseases, but rather a contributing factor in primarily acquired diseases. This hypothesis is supported by previous studies which have shown that family history of sudden death is a significant risk factor for sudden death also in middle-age and beyond, although cardiac diseases leading to SCD in this age group are primarily considered to be acquired^27, 28^. According to a recent study from Khera et al., very rare disease-causing variants are associated with only 2.5% of SCDs in general population^29^, which is unlikely to explain the inherited predisposition to SCDs in general population alone. The concept of disease-modifying polymorphisms has gained attention as an explanation to the variable SCD risk in patients with the same cardiac disease. Evidence for this has been published among patients with long QT syndrome^30^. To our knowledge, the present study is the first to investigate the role of rare genetic variants in SCDs that are due to acquired non-ischemic myocardial diseases. Although the difference in the prevalence of suspected variants was not statistically significant, disease-modifying rare gene variants in acquired myocardial diseases is potentially an important field for further research, especially because such cardiac diseases may be preventable with appropriate pharmacological treatments and lifestyle interventions.

## Limitations

First limitation is the absence of evidence regarding the co-segregation between the variants and the disease among first-degree family members. It is unreasonable to establish causality between variants and the disease solely based on the NGS study and current data regarding variant pathogenicity is incomplete. Our NGS sequencing method was neither able to detect copy number variation. Also, detection of myocardial fibrosis was based on subjective evaluation of macroscopic dissection and histological samples taken from 3 to 5 sections of the heart, which may not always be sufficient to detect scattered mild fibrosis. Although the Fingesture study includes the highest number of autopsy confirmed SCD cases in the world to our knowledge, we cannot be fully assured in every case that the SCD was actually due to the disease. Our study population was too small to generate statistically significant results, and further studies are needed to assess more precisely the impact of rare genetic variants on the occurrence of SCD among patients with hypertension and/or obesity related hypertrophic heart disease.

## Conclusions

Potentially disease-related rare variants in myocardial structure encoding genes are common among non-ischemic SCD victims with hypertension and/or obesity related hypertrophy and fibrosis at autopsy. Variants were mainly observed in genes related to HCM, ACM and DCM. Subjects with potentially disease-related variants had lower mean BMI and heart weight without significant difference in myocardial fibrosis. Taken into notice that such variants were also present in a small number of subjects with hypertension and cardiac hypertrophy, the results require further investigation as genetic modification may be responsible for the pattern of disease observed at autopsy in subjects with LVH associated with hypertension or obesity. The clinical relevance of this hypothesis lies in the importance of post-mortem investigations and in the guidance and treatment of genetically vulnerable subjects and their family members.

## Supplementary Information

Supplementary Information.

## Data Availability

All data generated or analysed during this study are included in this published article and its Supplementary Information files.
