# Supplementary material for: Genetic contributions to the expression of acquired causes of cardiac hypertrophy in non-ischemic sudden cardiac death victims
Source: Sci Rep. 2021 May 27;11:11171. doi: 10.1038/s41598-021-90693-7 (PMC8159951; doi:10.1038/s41598-021-90693-7)
Supplement: Supplementary file 1 — Supplementary Information. [file 41598_2021_90693_MOESM1_ESM.docx]

**Genetic Contributions to the Expression of Acquired Causes of Cardiac Hypertrophy in Non-Ischemic Sudden Cardiac Death Victims**

Lauri HOLMSTRÖM^a^, MD, PhD; Katri PYLKÄS^b^, PhD; Anna TERVASMÄKI^b^, PhD; Juha VÄHÄTALO^a^, BM; Katja PORVARI^c^, PhD; Lasse PAKANEN^c,d^, MD, PhD; Kari S KAIKKONEN^a^, MD, PhD; Juha S PERKIÖMÄKI^a^, MD, PhD; Antti M KIVINIEMI^a^, PhD; Risto KERKELÄ^e^, MD, PhD; Olavi UKKOLA^a^, MD, PhD; Robert J MYERBURG^f^, MD, PhD; Heikki V HUIKURI^a^, MD, PhD and Juhani JUNTTILA^a^, MD, PhD

^a^Research Unit of Internal Medicine, Medical Research Center Oulu, University of Oulu and Oulu University Hospital, Oulu, Finland

^b^Laboratory of Cancer Genetics and Tumor Biology, Cancer and Translational Medicine Research Unit and Biocenter Oulu, University of Oulu, Oulu, Finland

^c^Department of Forensic Medicine, Research Unit of Internal Medicine, Medical Research Center Oulu, University of Oulu, Oulu, Finland

^d^Forensic Medicine Unit, National Institute for Health and Welfare (THL), Oulu, Finland

^e^Research Unit of Biomedicine, University of Oulu, Oulu, Finland

^f^Division of Cardiology, University of Miami Miller School of Medicine, Miami, FL, USA (supported in part by the AHA Chair in Cardiovascular Research and a grant from the Miami Heart Research Institute; prior support from the Leducq Foundation, Paris, France, contributed to this work)

| **Patient no.** | **Mutated gene** | **Nucleotide change** | **Effect on protein** | **Predicted effect** | **NGS coverage** | **ExAC**  **>3,000 Finnish controls MAF** | \| **SISu >10,000 Finnish**  **controls MAF** \|  \|  \| \| --- \| --- \| --- \| \|  \|  \| | \| **ClinVar adjudication** \|  \|  \| \| --- \| --- \| --- \| \|  \|  \| |
| --- | --- | --- | --- | --- | --- | --- | --- | --- | --- | --- | --- | --- | --- | --- | --- | --- | --- | --- |
| 3 | *LMNA* | 1978A>G | Asn660Asp | Missense | 258 | 0.0001 | 0.0009 | Conflicting |
| 7 | *PKP2* | 1460A>G | Gln487Arg | Missense | 18 | Not detected | Not detected | VUS |
| 8 | *ACTN2* | 1426G>A | Ala476Thr | Missense | 91 | 0.0002 | 0.0001 | Conflicting |
|  | *LAMA4* | 1418T>C | Val473Ala | Missense | 284 | Not detected | Not detected | N/A |
|  | *MYH6* | 4906C>T | Arg1636Cys | Missense | 247 | Not detected | Not detected | Conflicting |
|  | *TTN* | 11254+2T>C |  | Affects canonical splicing | 78 | Not detected | Not detected | Conflicting |
| 10 | *DSG2* | 1643G>A | Arg548His | Missense | 108 | Not detected | Not detected | Conflicting |
| 11 | *DTNA* | 1099C>T | Pro367Ser | Missense | 160 | Not detected | Not detected | N/A |
| 12 | *DSC2* | 1559T>C | Ile520Thr | Missense | 47 | 0.0012 | 0.0016 | Benign |
|  | *NEXN* | 968A>G | Glu323Gly | Missense | 106 | 0.0021 | 0.0017 | N/A |
| 14 | *LMNA* | 1978A>G | Asn660Asp | Missense | 88 | 0.0001 | 0.0009 | Conflicting |
| 16 | *ACTN2* | 949C>A | Gln317Lys | Missense | 11 | Not detected | Not detected | N/A |
| 17 | *ACTN2* | 920G>T | Arg307Leu | Missense | 12 | Not detected | Not detected | N/A |
| 18 | *ANKRD1* | 820T>C | Tyr274His | Missense | 132 | 0.0004 | 0.0009 | Conflicting |
| 19 | *ANKRD1* | 820T>C | Tyr274His | Missense | 224 | 0.0004 | 0.0009 | Conflicting |
| 20 | *CASQ2* | 874G>T | Ala292Ser | Missense | 24 | 0.0020 | 0.0023 | Conflicting |
| 21 | *CSRP3* | 299G>A | Arg100His | Missense | 30 | 0.0053 | 0.0046 | Conflicting |
| 22 | *CTF1* | 382C>A | Gln128Lys | Missense | 12 | Not detected | Not detected | N/A |
| 23 | *CTF1* | 121G>A | Ala41Thr | Missense | 53 | Not detected | Not detected | N/A |
|  | *LDB3* | 466G>A | Ala156Thr | Missense | 46 | 0.0075 | 0.0076 | Benign |
|  | *MYLK2* | 1583G>A | Arg528Gln | Missense | 90 | Not detected | Not detected | N/A |
| 24 | *DES* | 665G>A | Arg222His | Missense | 234 | 0.0005 | 0.0010 | Conflicting |
| 25 | *DSG2* | 337G>A | Val113Ile | Missense | 12 | Not detected | Not detected | N/A |
| 26 | *DSG2* | 473T>G | Val158Gly | Missense | 56 | 0.0054 | 0.0079 | Benign |
| 27 | *DSP* | 1444T>G | Cys482Gly | Missense | 103 | 0.0002 | 0.0003 | N/A |
| 28 | *DSP* | 1043A>G | Glu348Gly | Missense | 12 | Not detected | Not detected | N/A |
| 29 | *DSP* | 6295-6296CC>AT | Pro2099Ile | Missense | 193 | 0.0035 | 0.0037 | VUS |
| 30 | *DTNA* | 92G>A | Arg31Gln | Missense | 95 | 0.0055 | 0.0056 | N/A |
| 31 | *DTNA* | 92G>A | Arg31Gln | Missense | 105 | 0.0055 | 0.0056 | N/A |
| 32 | *LAMA4* | 5232G>T | Gln1744His | Missense | 19 | Not detected | Not detected | N/A |
|  | *RYR2* | 9190G>A | Ala3064Thr | Missense | 52 | 0.0001 | 0.0008 | VUS |
| 33 | *LAMA4* | 2098G>A | Gly700Arg | Missense | 49 | Not detected | Not detected | N/A |
| 34 | *LAMA4* | 3218C>T | Thr1073Ile | Missense | 12 | Not detected | Not detected | N/A |
|  | *LAMA4* | 2461C>T | Leu821Phe | Missense | 28 | Not detected | Not detected | N/A |
| 35 | *LAMA4* | 3054G>T | Leu1018Phe | Missense | 186 | Not detected | Not detected | Conflicting |
| 36 | *LDB3* | 1385C>A | Pro462Gln | Missense | 23 | Not detected | Not detected | N/A |
|  | *MYLK2* | 902C>A | Thr301Asn | Missense | 65 | 0.0022 | 0.0031 | VUS |
| 37 | *LDB3* | 466G>A | Ala156Thr | Missense | 82 | 0.0075 | 0.0076 | Benign |
| 38 | *LDB3* | 466G>A | Ala156Thr | Missense | 72 | 0.0075 | 0.0076 | Benign |
| 39 | *LDB3* | 466G>A | Ala156Thr | Missense | 164 | 0.0075 | 0.0076 | Benign |
| 40 | *LDB3* | 566C>T | Ser189Leu | Missense | 65 | 0.0001 | 0.0009 | Conflicting |
| 41 | *LDB3* | 919G>A | Val307Ile | Missense | 73 | 0.0002 | 0.0005 | N/A |
| 42 | *MYH6* | 817C>T | Arg273Trp | Missense | 20 | Not detected | Not detected | N/A |
| 43 | *MYH6* | 2807C>T | Ala936Val | Missense | 257 | 0.0047 | 0.0053 | N/A |
| 44 | *MYH6* | 2807C>T | Ala936Val | Missense | 172 | 0.0047 | 0.0053 | N/A |
| 45 | *MYH6* | 2807C>T | Ala936Val | Missense | 175 | 0.0047 | 0.0053 | N/A |
| 46 | *MYH7* | 4186C>T | Arg1396Trp | Missense | 16 | Not detected | Not detected | VUS |
| 47 | *NEXN* | 968A>G | Glu323Gly | Missense | 145 | 0.0021 | 0.0017 | N/A |
| 48 | *PKP2* | 419C>T | Ser140Phe | Missense | 383 | 0.0029 | 0.0014 | Conflicting |
| 49 | *PKP2* | 1460A>G | Gln487Arg | Missense | 25 | Not detected | Not detected | VUS |
| 50 | *PKP2* | 1490G>A | Arg497Gln | Missense | 27 | Not detected | Not detected | Conflicting |
| 51 | *RBM20* | 3412G>A | Glu1138Lys | Missense | 30 | Not detected | Not detected | N/A |
| 52 | *RBM20* | 3373G>A | Glu1125Lys | Missense | 130 | 0.0012 | 0.0017 | Benign |
| 53 | *RBM20* | 1958C>T | Thr653Ile | Missense | 90 | Not detected | 0.0008 | Conflicting |
|  | *SGCD* | 475G>A | Val159Ile | Missense | 17 | Not detected | Not detected | VUS |
| 54 | *RYR2* | 6433G>C | Gly2145Arg | Missense | 175 | 0.0002 | 0.0002 | VUS |
| 55 | *RYR2* | 9820A>G | Asn3274Asp | Missense | 202 | 0.0014 | 0.0016 | VUS |
| 56 | *RYR2* | 9190G>A | Ala3064Thr | Missense | 101 | 0.0001 | 0.0008 | VUS |
| 57 | *VCL* | 2638G>A | Glu880Lys | Missense | 12 | Not detected | Not detected | N/A |

***Supplementary table 1.*** Variants of uncertain significance among sudden cardiac death victims with presumed acquired disease at autopsy. MAF=Minor allele frequency, NGS=Next generation sequencing, N/A=Not Available, VUS=Variant of Uncertain Significance.

| **Likely pathogenic variants/Patient number** | **Mutated gene** | **Nucleotide change** | **Effect on protein** | **Predicted effect** | **NGS coverage** | **ExAC**  **>3,000 Finnish controls MAF** | \| **GnomAD>10,000**  **Finnish**  **controls MAF** \| \| --- \| \| | **ACMG score** |
| --- | --- | --- | --- | --- | --- | --- | --- | --- | --- |
| 1 | *LDB3* | 655C>T | Arg219Ter | Truncating | 166 | Not detected | Not detected | ***PVS1 + PM2*** |
| 2 | *MYBPC3* | 2555dupT | Gly853ArgfsTer31 | Frameshift | 317 | Not detected | 0.0001 | ***PVS1 + PM2*** |
| **VUS/Patient number** |  |  |  |  |  |  |  |  |
| 3 | *CSRP3* | 299G>A | Arg100His | Missense | 350 | 0.0053 | 0.0046 |  |
| 4 | *DES* | 200G>C | Gly67Ala | Missense | 369 | Not detected | Not detected |  |
| 5 | *DSP* | 6295-6296CC>AT | Pro2099Ile | Missense | 774 | 0.0035 | 0.0037 |  |
| 6 | *DTNA* | 92G>A | Arg31Gln | Missense | 257 | 0.0055 | 0.0056 |  |
| 7 | *FHL2* | 298A>G | Lys100Glu | Missense | 191 | Not detected | Not detected |  |
| 8 | *LAMA4* | 2594T>C | Met865Thr | Missense | 194 | 0.0001 | 0.0001 |  |
|  | *RBM20* | 1958C>T | Thr653Ile | Missense | 194 | Not detected | 0.0008 |  |
| 9 | *LDB3* | 566C>T | Ser189Leu | Missense | 139 | 0.0001 | 0.0009 |  |
| 10 | *LDB3* | 566C>T | Ser189Leu | Missense | 122 | 0.0001 | 0.0009 |  |
| 11 | *MYH6* | 2807C>T | Ala936Val | Missense | 673 | 0.0047 | 0.0053 |  |
| 12 | *NEXN* | 968A>G | Glu323Gly | Missense | 483 | 0.0021 | 0.0017 |  |
| 13 | *RBM20* | 1958C>T | Thr653Ile | Missense | 270 | Not detected | 0.0008 |  |
| 14 | *RYR2* | 9190G>A | Ala3064Thr | Missense | 209 | 0.0001 | 0.0008 |  |

***Supplementary table 2.*** Non-benign variants among control patients with hypertension and hypertrophic heart on 20 years of follow-up.
